# Etoposide (VP-16)-Containing Oral Palliative Chemotherapies (OPCs) Are Well Tolerated by Elderly Patients With Aggressive Non-Hodgkin Lymphoma: A 10-Year Single-Institute Experience

**DOI:** 10.7759/cureus.92321

**Published:** 2025-09-14

**Authors:** Kenichiro Takeda, Yusuke Yamaga, Shoichiro Okazaki, Takahiro Nishiyama

**Affiliations:** 1 Hematology, Ichinomiya Municipal Hospital, Ichinomiya, JPN

**Keywords:** aggressive lymphoma, elderly nhl, etoposide therapy, non-hodkins lymphoma, oral regimens, palliative chemotherapy, quality of life (qol), real-world outcomes

## Abstract

Background: Non-Hodgkin lymphoma (NHL) is a malignant neoplasm with lymphoid-cell origin. Some patients are refractory to the treatments. Not all patients with relapsed/refractory NHL can benefit from intensive salvage therapy and novel drugs. Oral palliative chemotherapies (OPCs) are potentially good options for these patients through the alleviation of the lymphoma-related symptoms and the ability to stay at home for longer durations.

Methods: Etoposide (VP-16)-containing OPCs are often used for aggressive NHL as palliative use at our institute. In order to elucidate the toxicity and efficacy of VP-16-OPCs, we retrospectively reviewed the patients at our institute with medical charts. We also reviewed the hospitalization-free period, indicating successful symptom palliation.

Results: Fifty-six patients who received VP-16-OPCs at our institute between April 2012 and December 2023 were reviewed. Diseases include adult T-cell leukemia/lymphoma (ATLL), diffuse large B-cell lymphoma (DLBCL), peripheral T-cell lymphoma (PTCL), and others. VP-16-OPCs include VENP, Sobuzoxane (MST)/VP-16, and VP-16+PSL (prednisolone). Infection was the most common non-hematological adverse event (34%). Nineteen out of 40 patients could complete more than two courses of VENP. Patients who responded to VP-16-OPCs tended to have longer event-free survival (EFS) (46 vs. 268 days, p < 0.01) and hospitalization-free periods (46 vs. 245 days, p = 0.11) compared to those who did not respond.

Conclusion: We here reported the largest Japanese real-world dataset on VP-16-OPCs. The VP-16-OPCs were well tolerated by the patients, and some of them benefited from the treatment. These regimens can be considered when intensive and novel therapies are not indicated.

## Introduction

Non-Hodgkin lymphoma (NHL) is a heterogeneous group of lymphoid neoplasms. The number of treatment options for relapse/refractory lymphomas has significantly expanded in recent years. Notably, chimeric antigen receptor T-cell therapies [1-3] have emerged as standard treatments for relapse/refractory diffuse large B-cell lymphoma (DLBCL). Additionally, novel molecular target drugs have been developed [4-7]. These advancements have the potential to improve outcomes for patients with relapse/refractory lymphomas.

Despite the development of these novel drugs, not all patients prefer the novel treatments because of comorbidities, treatment-related adverse effects, and poor accessibility. Particularly, elderly frail patients often prefer the best supportive care to remain at home for longer periods.

From another perspective, progressive NHL is associated with significantly higher health care costs. Safe and efficient palliative treatments are required from a health economic perspective [8].

Over the past decade, our institute has used different Etoposide (VP-16) containing oral palliative chemotherapies (OPCs) for palliative care in order to alleviate symptoms related to lymphoma, such as fever, pain, and fatigue. The three regimens used were as follows: VENP, which comprises VP-16, cyclophosphamide (CPA), procarbazine (PCZ), and prednisolone (PSL); Sobuzoxane (MST)/VP-16, which is composed of MST and VP-16; and VP-16+PSL.

Previous studies have reported other OPCs, including dexamethasone, etoposide, chlorambucil, lomustine (DECC) [9], lomustine, etoposide, and prednisone (CEP) [10], and COCKLE [11]. However, the efficacy and tolerability of these agents remain controversial. Moreover, these regimens include lomustine, which is not approved for malignant lymphoma in the Japanese insurance system. The prednisolone, etoposide, procarbazine, cyclophosphamide (PEP-C) regimen is well reported in these settings and utilizes the same anticancer drugs [12]. However, its protocol is not precisely the same as with our VP-16-OPCs and is not validated in Japanese populations.

The aim of the study is to evaluate the tolerability and efficacy of VP-16-OPCs for aggressive NHL over the past decade.

## Materials and methods

Ethics approval

The clinical study design was reviewed and approved by the Ethics Committee of Ichinomiya Municipal Hospital (IRB No. 1453-A2025068). The current study was performed in accordance with the Declaration of Helsinki. Informed consent was obtained using an opt-out system.

Patients

Patients who received VP-16-OPCs at Ichinomiya Municipal Hospital between April 2012 and December 2023 were identified via a retrospective chart review. Data analysis was performed after IRB approval. The inclusion criteria for the present study were as follows: aged over 20 years with a pathologically confirmed diagnosis of aggressive NHL at the Ichinomiya Municipal Hospital. Exclusion criteria were the presence of a prior or concurrent second malignancy or central nervous system (CNS) involvement.

Treatment schedule

The schedule of the VENP regimen was as follows: VP-16 50 mg once a day, CPA 50 mg once a day, PCZ 50 mg once a day, and PSL 30 mg once a day. The MST/VP-16 schedule was as follows: MST 400 mg once or twice a day and VP-16 50 mg once a day. In both regimens, the drugs were administered from days 1 to 14. Each treatment cycle was 21 days.

The VP-16+PSL regimen was as follows: VP-16 was started at a dose of 25-150 mg and adjusted according to the degree of bone marrow suppression. PSL was continuously administered at a dose of 10-30 mg.

Patient evaluation

The patients’ medical records were reviewed to conduct an evaluation. The radiological tumor assessments were performed based on the physician’s discretion. Responses were assessed according to the response evaluation criteria in solid tumors (RECIST) version 1.0. Toxicity was evaluated according to the Common Terminology Criteria for Adverse Events version 4.0. Events were described as any undesirable outcomes, including death, tumor progression, and treatment discontinuation, attributed to adverse events. We collected the overall response (ORR), overall survival (OS), event-free survival (EFS), and cumulative incidence of adverse events. Chemoresistance was defined as complete remission for <1 year with the prior regimen. Chemosensitivity was defined as remission for ≥1 year.

Statistical analysis

Clinical and laboratory data were analyzed using EZR for Mac (R version 4.4.0). Continuous variables were compared using the Mann-Whitney U test, and nominal variables were compared using the chi-square (Χ2) test. The cumulative incidence was evaluated using Gray’s test. The overall survival was analyzed with the Kaplan-Meier method and assessed with the log-rank test. In all analyses, a p-value of <0.05 was considered statistically significant.

## Results

Characteristics of the patients

Fifty-six patients received VP-16-OPCs. Forty-nine patients with relapse/refractory disease were indicated for VP-16-OPCs, and the other seven patients with an unfit physical status were treated with first-line therapy. Twenty-five patients were women. The median age of all patients was 76.5 (range: 50-94) years. The VP-16-OPCs were as follows: VENP, n = 32; MST/VP-16, n = 4; VENP and MST/VP-16, n = 8; and VP-16+PSL, n = 12 (Table 1).

**Table 1:** Patients’ characteristics ATLL: Adult T-cell Leukemia/Lymphoma, DLBCL: Diffuse Large B-cell Lymphoma, PTCL: Peripheral T-cell Lymphoma, IPI: International Prognostic Index

Factor	Group	Overall (n=56)
Median age, years (range)		76.5 (50–94)
Age, n (%)	≤65	6 (10.7)
	66–74	15 (26.8)
	≥75	35 (62.5)
Sex, n (%)	F	25 (44.6)
	M	31 (55.4)
Lymphoma subtypes, n (%)	ATLL	5 (8.9)
	DLBCL	35 (62.5)
	PTCL	7 (12.5)
	Other	9 (16.1)
ECOG performance status, n (%)	0–2	48 (85.7)
	3–4	8 (14.3) all hospitalized
Ann Arbor stage, n (%)	III-IV	56 (100)
IPI, n (%)	0-2	22 (40.0)
	≥3	32 (58.2)
	NA	1 (1.8)
Prior therapy lines, n (%)	≤2	22 (39.3)
	3	16 (28.6)
	≥4	18 (32.1)
Regimen, n (%)	MST/VP-16	4 (7.1)
	VENP	32 (57.1)
	VENP, MST/VP-16	8 (14.3)
	VP16+PSL	12 (21.4)

In the VENP regimen, the drugs were administered for a median of seven (3-14) days with an interval of four (3-8) weeks at the start and a median of seven (3-14) days with an interval of four (3-8) weeks at the end of the treatment. Dose reduction or interval modification was performed in 12 patients. Nine patients used rituximab.

In the MST/VP-16 regimen, the drugs were administered for a median of four (2-5) days with an interval of four weeks at the initiation and four (3-5) days with an interval of four (3-4) weeks at the end. One patient had a dose reduction.

In the VP-16+PSL regimen, the drugs were administered for a median of seven (3-10) days with an interval of four (2-4) weeks and 8.5 (7-10) days with an interval of four (3-4) weeks. None of the patients had a dose reduction.

Further, 19 of 40 patients completed greater than two courses of VENP. However, 11 of 15 patients treated with MST/VP-16 and 6 of 12 who received VP-16+PSL only had one or two courses of treatment. The reasons for treatment discontinuation were as follows: disease progression, n = 43; patient’s preference, n = 2; and adverse events, n = 7. There were 46 recorded deaths, and the causes were disease progression (n = 43) and infection (n = 3).

Overall responses, event-free survival, and overall survival

The ORR was 23% (13 patients), consisting of a 3% complete response (CR) rate and a 20% partial response (PR) rate (Table 2). 

**Table 2:** Adverse events Response rates of each oral palliative chemotherapy (VP-OPCs). The effects of chemoresistance and chemosensitivity are also described. CR: Complete Response, PR: Partial Response, ORR: Overall Response Rate, SD: Stable Disease, PD: Progressive Disease

Response	% (n=56)	VENP % (n=32)	MST/VP-16 % (n=4)	VENP, MST/VP-16 % (n=8)	VP-16 % (n=12)	Chemoresistant % (n=45)	Chemosensitive % (n=11)
CR	3 (2)	6 (2)	0	0	0	2 (1)	9 (1)
PR	20 (11)	16 (5)	0	25 (2)	33 (4)	20 (9)	18 (2)
ORR	23 (13)	22 (7)	0	25 (2)	33 (4)	22 (10)	27 (3)
SD	45 (25)	48 (15)	50 (2)	50 (4)	33 (4)	44 (20)	45 (5)
PD	32 (18)	30 (10)	50 (2)	25 (2)	33 (4)	36 (15)	28 (3)

**Figure 1:**
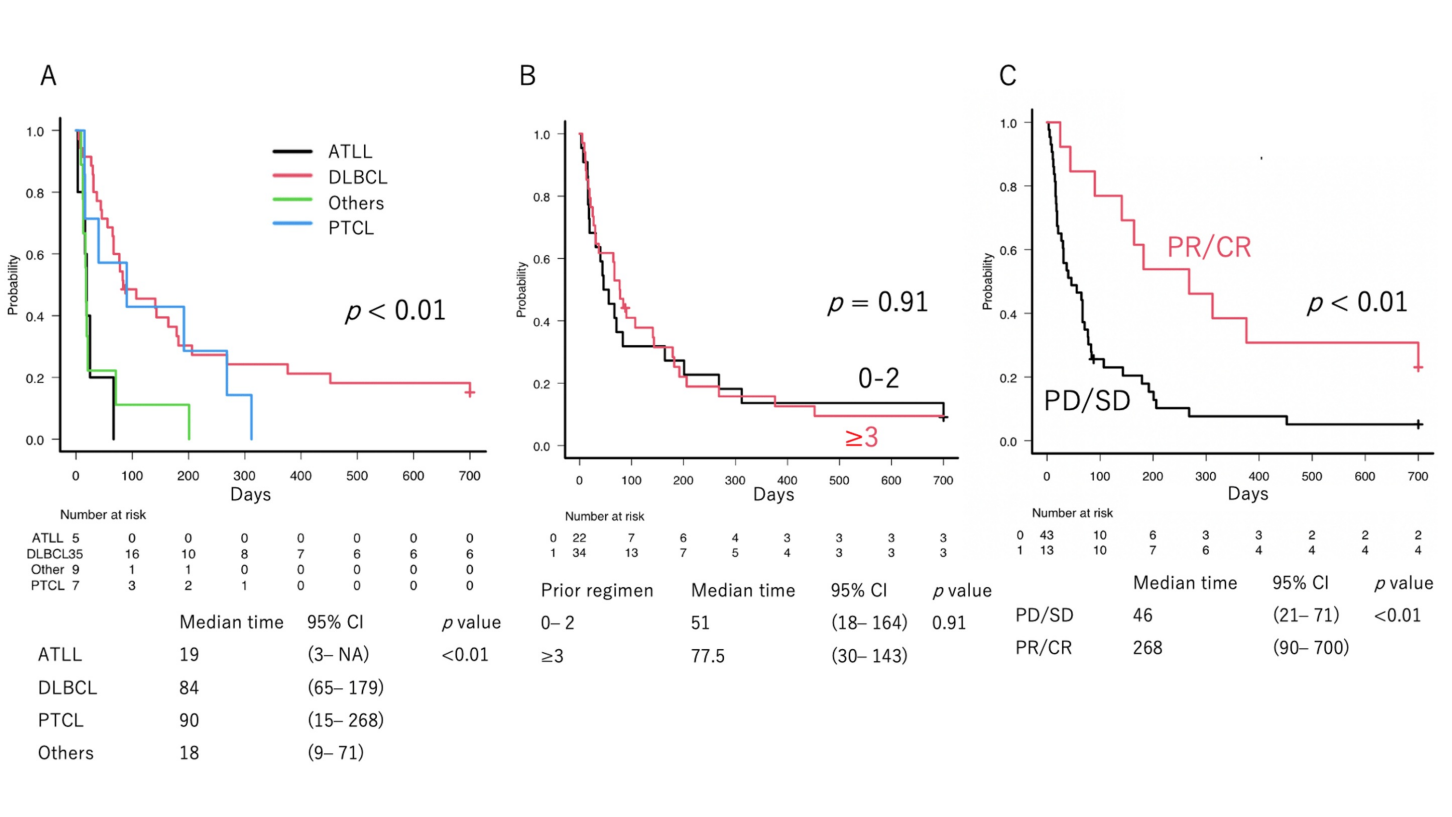
Event-free survival Event-free survival (EFS) since VP-16-containing oral palliative care treatment initiation. (A) Disease histological type significantly affected the EFS; (B) Prior treatment did not demonstrate a significant association with EFS; (C) Response to VP-16-containing treatment was significantly related to longer EFS.

**Figure 2:**
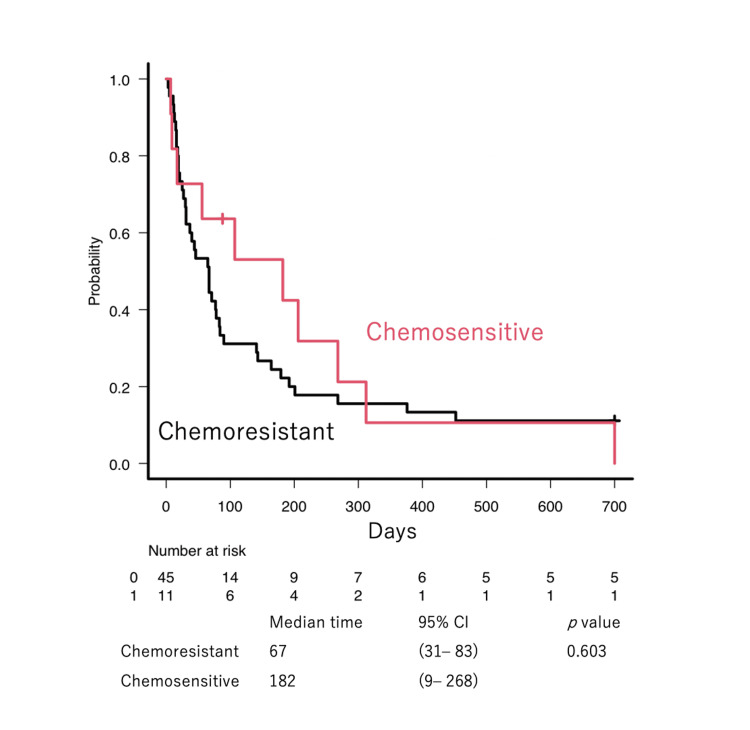
EFS by first CR duration Event-free survival (EFS) by stratified by chemosensitivity. Patients with a complete remission (CR) duration of more than one year were chemosensitive, while those with a CR duration of less than one year were classified as chemoresistant.

The median EFS days after the initiation of VP-16-OPCs was 67 days. In particular, patients with ATLL had inferior outcomes compared with those with DLBCL and PTCL (Figure 1A, p < 0.01). Not surprisingly, patients who achieved ORR exhibited a prolonged EFS (Figure 1C, 46 vs. 268 days, p < 0.01). Prior treatment response did not affect the EFS (Figure 2).

**Figure 3:**
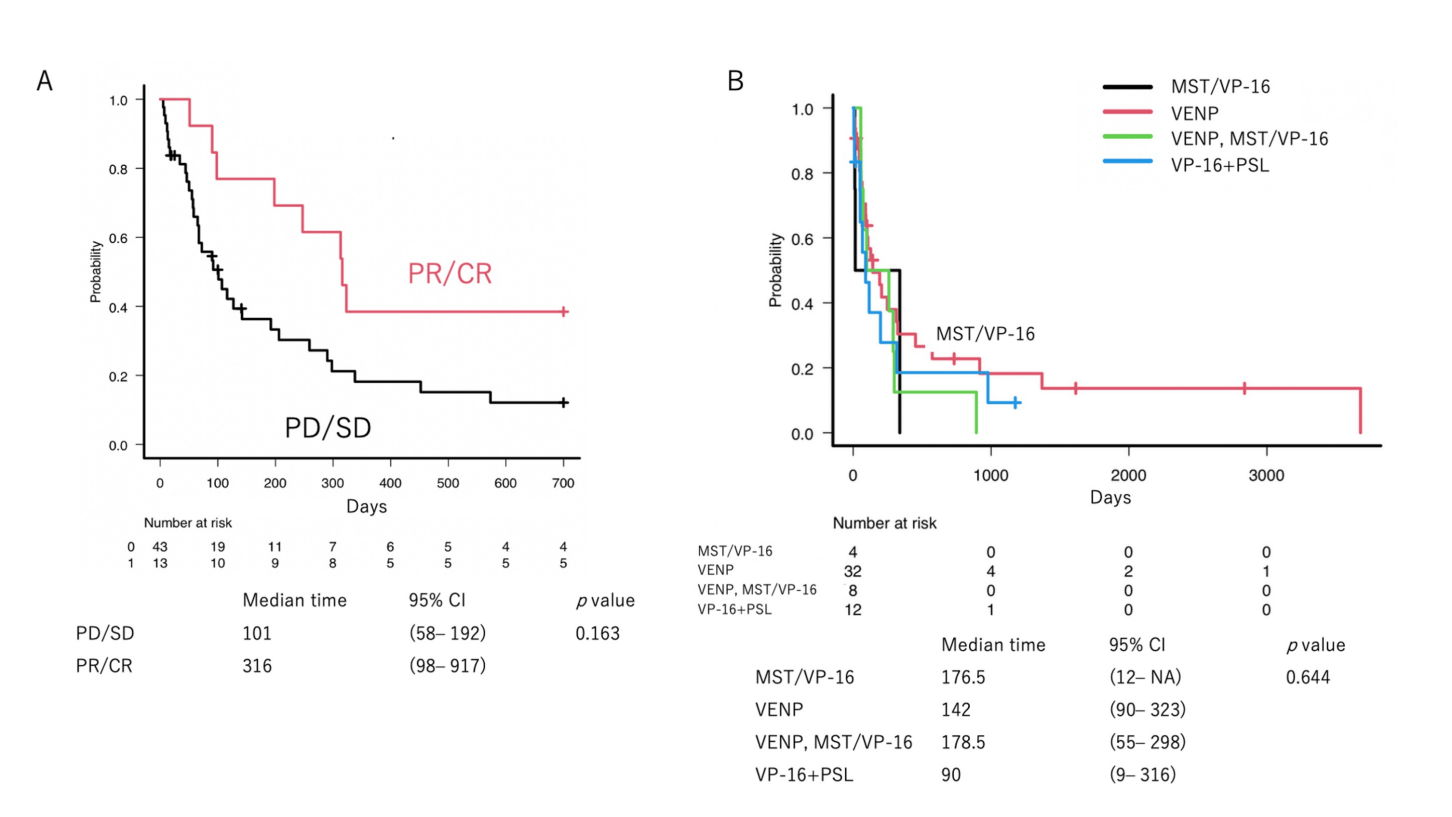
Overall survival Overall survival (OS) since the initiation of VP-16-containing oral palliative care treatment. (A) A superior response (PR/CR) to VP-16-containing treatment did not have a significant impact on OS. (B) No clear preference was observed among the VP-16-containing regimens.

The median survival period was 127 days. The median observation period for the patients who survived was 696.5 days. There was no statistically significant difference in OS by response rates (Figure 3A, *p* = 0.16) and treatment regimens (Figure 3B, *p* = 0.64).

Toxicity: hematologic and non-hematologic adverse events

Grade 3 or higher hematological adverse events did not significantly differ according to the VP-16 dose. In terms of non-hematological adverse events, infection was the most common adverse event; 19 patients presented with infections (Table 3).

**Table 3:** Adverse events Incidence rates of the Grade ≥3 hematological and non-hematological AEs are shown. AE: Adverse events, GI bleeding: Gastrointestinal bleeding

Grade ≥3 Hematological AEs	% (n=56)	VENP % (n=32)	MST/VP-16 % (n=4)	VENP, MST/VP-16 % (n=8)	VP-16 % (n=12)
						Neutropenia	48 (27)	60 (19)	50 (2)	50 (4)	13 (2)
Anemia	25 (14)	31 (10)	0	13 (1)	19 (3)
Thrombocytopenia	30 (17)	34 (11)	50 (2)	25 (2)	13 (2)
Grade ≥3 non-Hematological AEs					
						Infection	34 (19)	38 (12)	25 (1)	38 (3)	19 (3)
Nausea	7 (4)	10 (3)	25 (1)	0	0
Ileus	5 (3)	10 (3)	0	0	0
Mucositis	9 (5)	3 (1)	0	13 (1)	19 (3)
GI bleeding	4 (2)	6 (2)	0	0	0

**Figure 4:**
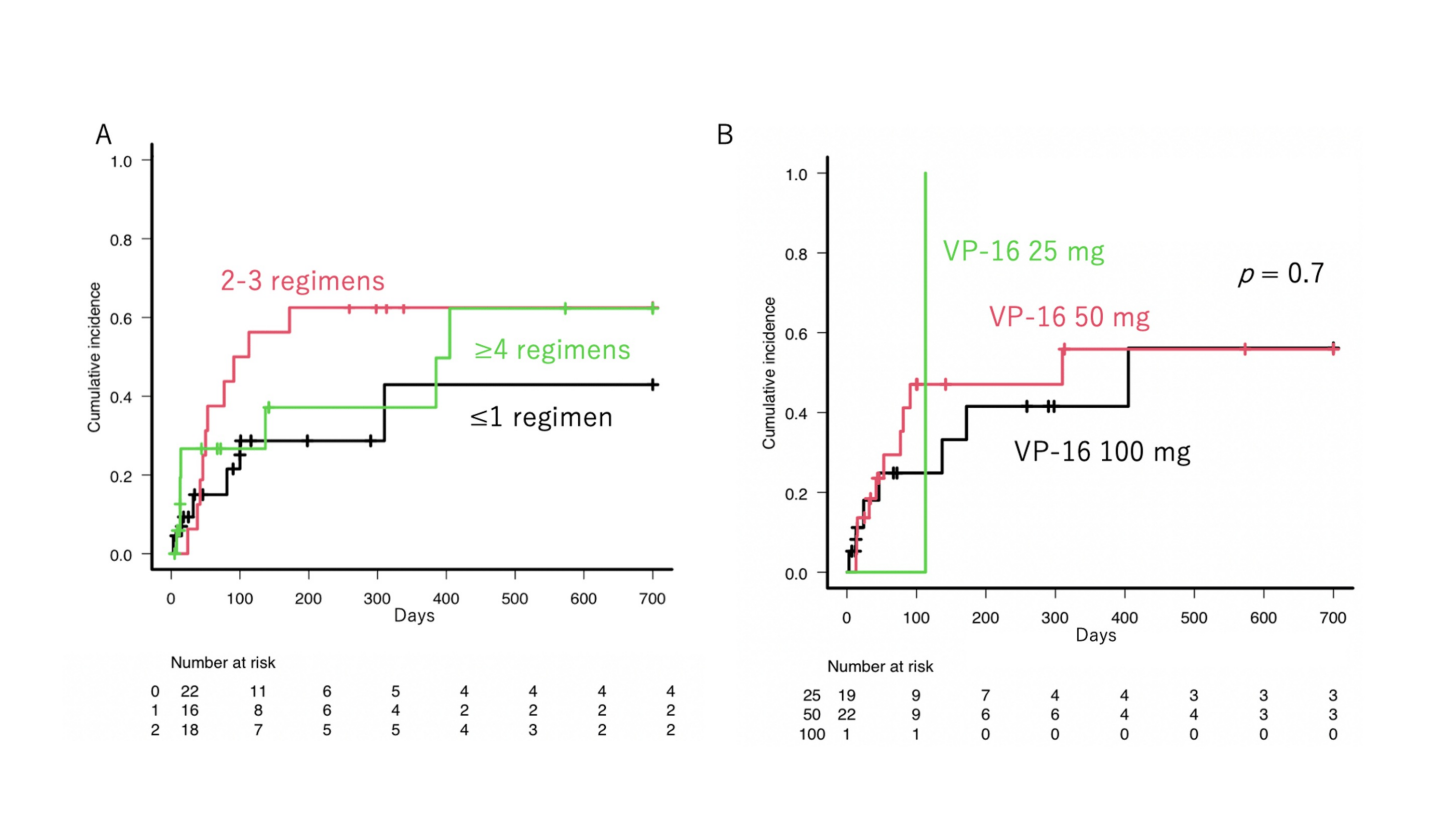
The cumulative incidence of febrile neutropenia The cumulative incidence of febrile neutropenia was described (A) according to the prior regimen courses and (B) according to the VP-16 doses.

The cumulative incidence (CI) rate of FN was similar between the previous treatment lines (Figure 4A, p = 0.5) and the dose of VP-16 (Figure 4B, p = 0.7).

**Figure 5:**
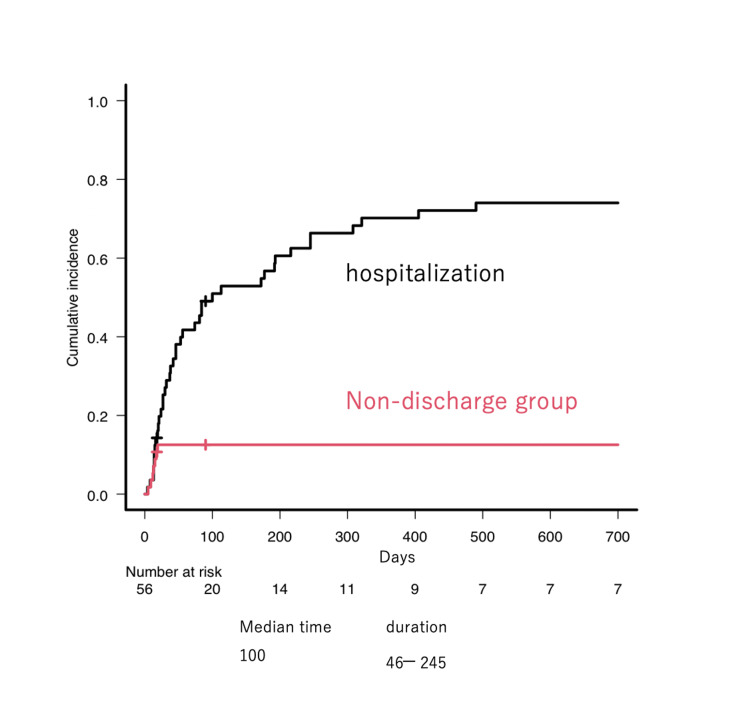
The cumulative incidence of hospitalization The cumulative incidence of hospitalization was shown. Some patients received VP-16-OPCs and were unable to be discharged from the hospital (classified as the non-discharge group)

**Figure 6:**
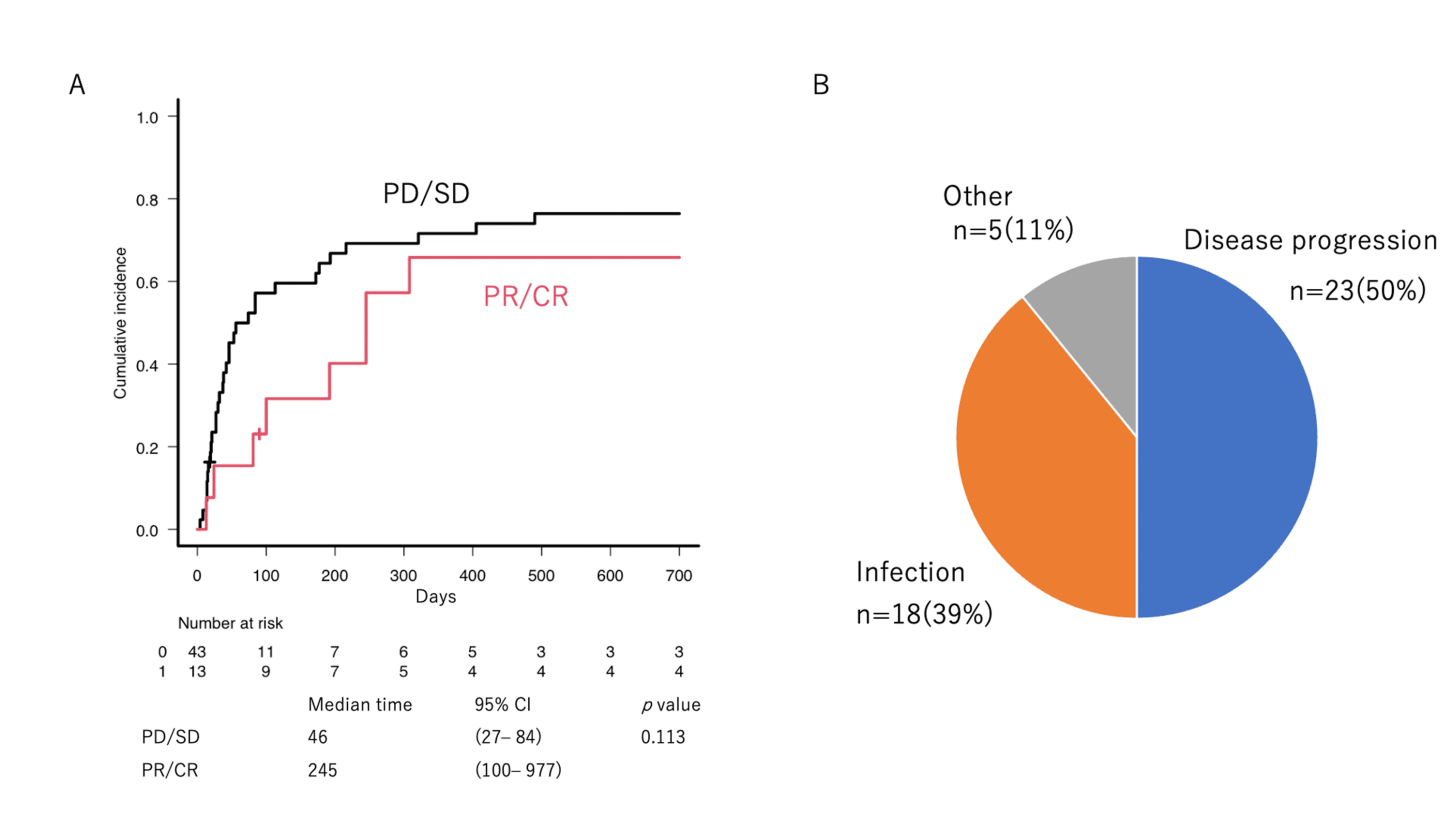
The cumulative incidence and cause of hospitalization (A) The cumulative incidence of hospitalization was described according to the response to VP-16-containing regimens. (B) The cause of hospitalization was shown.

To assess how long patients could remain at home, the CI of hospitalization was evaluated. The median hospitalization-free period was 100 (46-245) days (Figure 5). Patients who achieved ORR were more likely to have a longer hospitalization-free duration (median hospitalization-free period: 46 vs. 245 days, p = 0.113, Figure 6A). The primary reasons for hospitalization were disease progression in 50% of patients, infection in 39%, and others in 11% (Figure 6B).

## Discussion

To our knowledge, this is the largest single-institute dataset from Asia documenting aggressive NHL cases treated with VP-16 OPCs over a decade. VP-16-OPC treatments were well tolerated, considering that the patients were not indicated for standard therapies. Treatment-related mortality was observed in three patients, and all of them had infections. In the majority, VP-16-OPCs were selected as the final regimen. Although the OS did not differ between the response and non-response groups, the incidence of events significantly decreased in the response group. The death was observed in 46 cases. Forty-three out of 46 were due to the disease progression, and the other three cases were infections (COVID-19, PCP, and FN). From this perspective, our VP-16-OPCs are expected to alleviate the symptoms of aggressive NHLs; however, the ultimate fatal fate cannot be changed by this treatment. Therefore, those patients may benefit from the VP-16-OPCs and can potentially remain at home for longer periods of time.

The most frequently observed AEs were infections. Given that 50% of hospitalizations were attributed to infections, prophylaxis is of significant importance. Granulocyte-colony stimulating factor (GCSF) administration and more aggressive prophylaxis can be the management options. This is because of 19 patients who had infectious complications; 11 had febrile neutropenia. The use of prophylactic antibiotics may be an option for patients with refractory infections [13].

Based on this observation study, we currently manage the VENP regimen as follows. Treatment is initiated with full-dose VENP treatment and followed up weekly. If the general weakness or cytopenias develop, VENP is discontinued at week two. If treatment is tolerated, it is continued up to two weeks. The next treatment schedule remains unchanged unless uncontrolled infections occur. In cases of neutropenia (absolute neutrophil count < 500/uL), empirical prophylaxis for FN prophylaxis with levofloxacin is initiated and continued until stable recovery of the bone marrow functions. If the bone marrow recovery has not occurred by the next scheduled initiation, dose reduction is considered. For severely frail patients, it is possible to perform a dose reduction from the first treatment course.

The PEP-C regimen is a similar OPC regimen, which comprises the same oral chemotherapeutic drugs (Table 4). Despite significant differences in patient characteristics, the treatment outcomes of VENP were nearly comparable to those of other salvage chemotherapeutic regimens. The ORR of our VENP therapy was 23%. Due to the racial and geographic differences, our study cohort included ATLL patients who showed worse outcomes. This may have affected the ORR of our study. 

**Table 4:** Other PEP-C reports Similar oral palliative chemotherapy (OPC) regimens were compared to evaluate differences in efficacy and safety. NHL: Non-Hodgkin Lymphoma, ORR: Overall Response Rate

Reference	n	Disease	ORR	discontinuation due to toxicity	Infection (grade 3 or higher)	marrow suppression
Our Study	56	Aggressive NHL	23%	16% discontinued	34%	25-48%
Morton Coleman et al., Cancer, 2008 [12]	75	NHL (including low grade lymphomas)	69%	13% discontinued	11%	Not described as adverse events
Morton Coleman et al., Hematology, 2012 [14]	122	NHL		Not described	5%	Not described as adverse events
Simon J Bulley et al., EJ Haem, 2022 [15]	92	NHL	33%	29% discontinued	39%	18%

Regarding AEs, patients with VENP had a higher incidence of grade 3 or higher infections. This may be due to the difference in dose adjustment strategies. PEP-C consists of two phases: the induction and maintenance phases. Additionally, dose adjustments were generally not performed, and the schedules of administration were adjusted. However, as reported, dose modifications are well performed in actual clinical settings [12,14,15].

The other OPCs that were reported are DECC, CEP, and COCKLE [9-11]. However, because lomustine is not approved in Japan, VP-16-OPCs are the only available OPCs in Japan [16].

**Figure 7:**
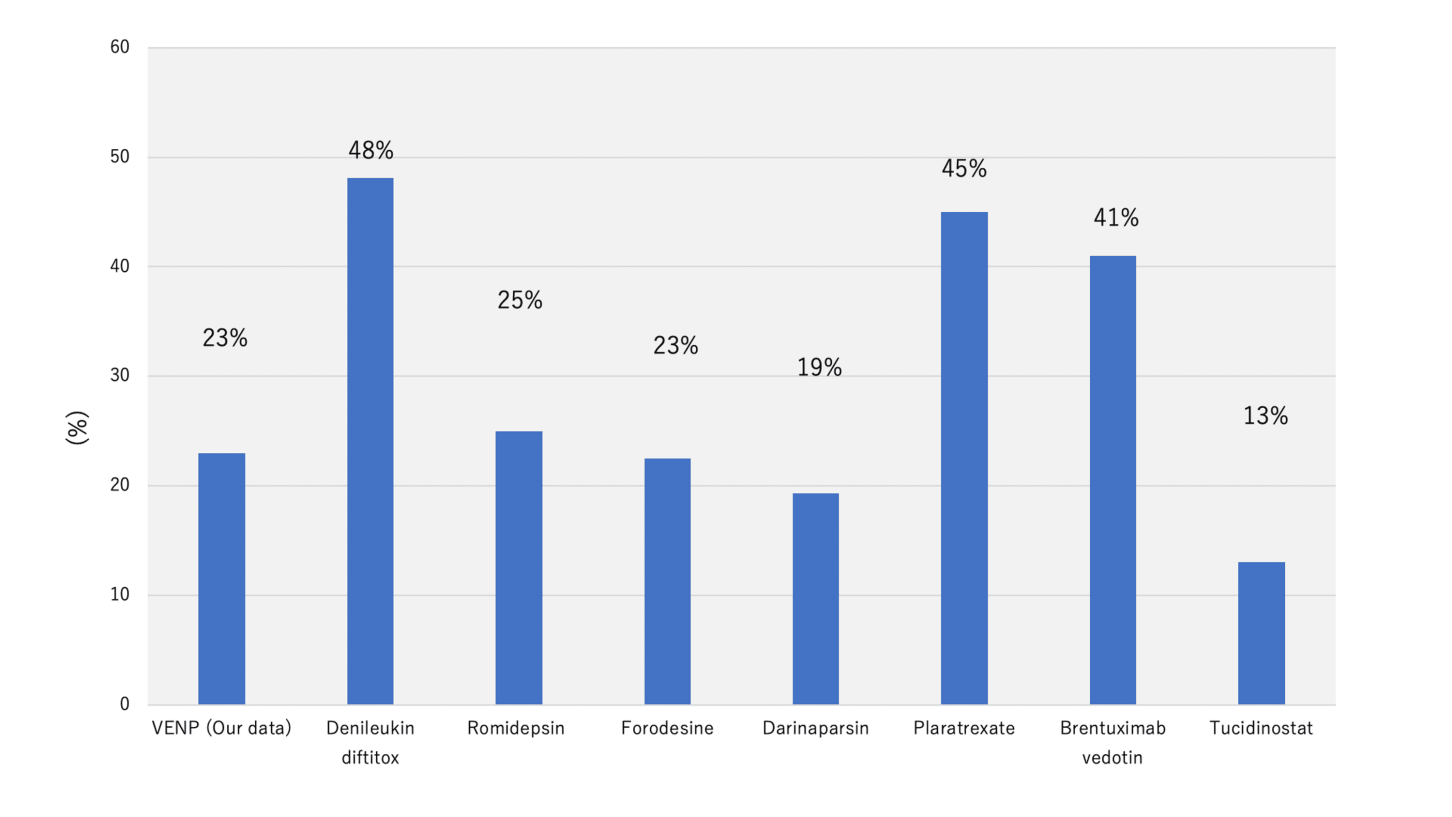
The comparison of overall response The image is created by the author. The overall response (ORR) of PTCL to each salvage regimen.

**Figure 8:**
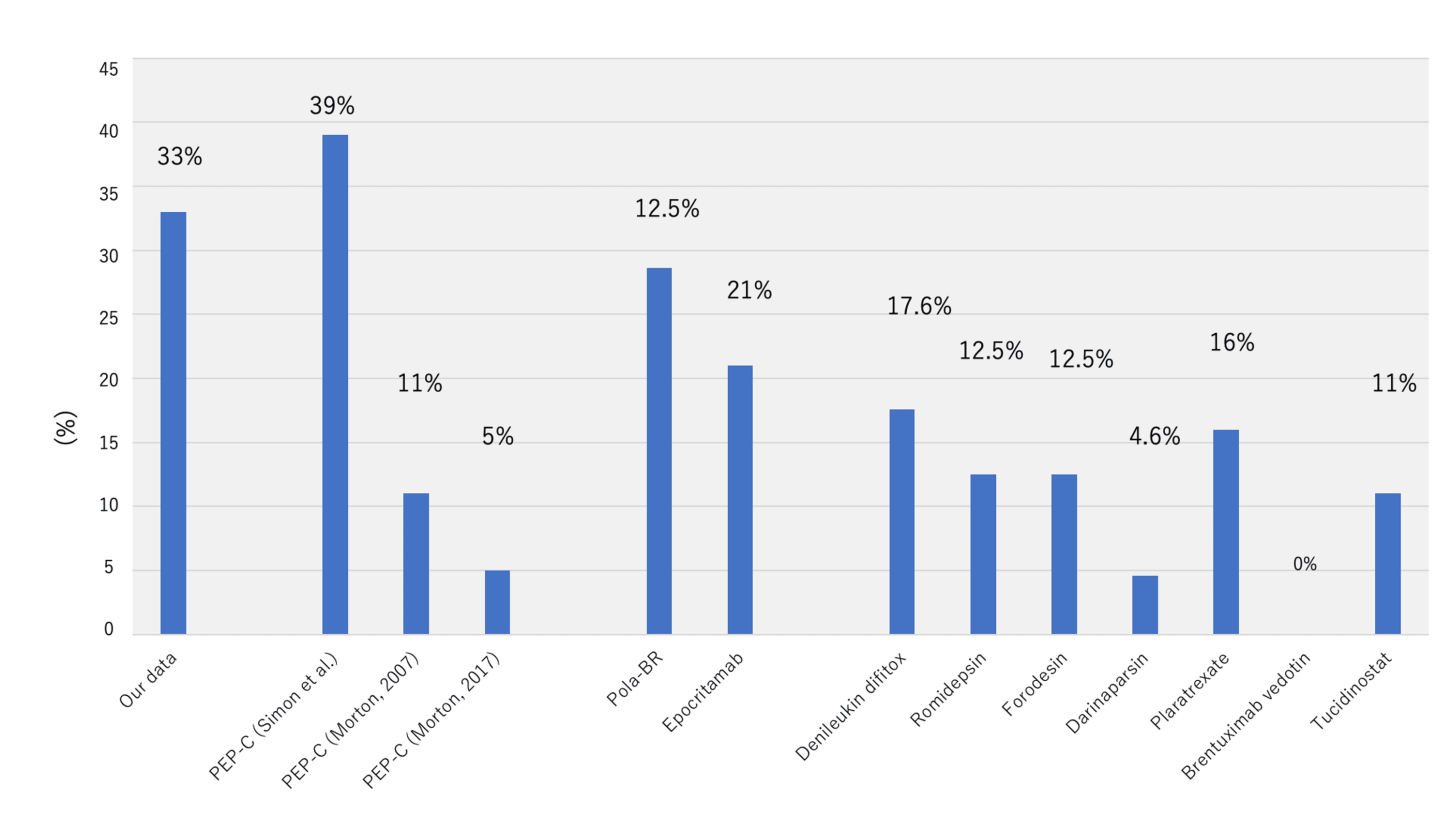
The comparison of infectious adverse events The incidence of infection (grade 3 or higher) was described. The image is created by the author.

Although the characteristics of the patients significantly differed, the treatment outcomes of VENP were almost comparable to those of other salvage chemotherapeutic regimens (Figures 7, 8) [5-7,12,14,17-20]. As for the drug prices, the cost significantly differed between VENP and the novel drugs. The difference in drug prices is directly related to the application of therapy, especially in low-resource settings, such as rural areas, and for frail elderly patients.

This study has several limitations. First, it is a single-institute report with a limited number of patients. Second, the treatment protocol was not strictly defined and was modified by each physician’s choice. Finally, the study was conducted as a single-arm investigation without a control group. To accurately assess the clinical benefit of our VP-16-OPCs, prospective studies will be necessary for confirmation.

## Conclusions

In conclusion, this study demonstrates that VP-16-OPCs are safe, effective, and economically viable for patients with aggressive NHL who are unsuitable for standard therapies. The favorable tolerability profile makes OPC a practical option for palliative care, particularly in resource-limited settings or for elderly and frail patients.

## References

[1] Bishop MR, Dickinson M, Purtill D (2022). Second-line tisagenlecleucel or standard care in aggressive B-cell lymphoma. N Engl J Med.

[2] Kamdar M, Solomon SR, Arnason J (2022). Lisocabtagene maraleucel versus standard of care with salvage chemotherapy followed by autologous stem cell transplantation as second-line treatment in patients with relapsed or refractory large B-cell lymphoma (TRANSFORM): results from an interim analysis of an open-label, randomised, phase 3 trial. Lancet.

[3] Locke FL, Miklos DB, Jacobson CA (2022). Axicabtagene ciloleucel as second-line therapy for large B-cell lymphoma. N Engl J Med.

[4] Iżykowska K, Rassek K, Korsak D, Przybylski GK (2020). Novel targeted therapies of T cell lymphomas. J Hematol Oncol.

[5] Piekarz RL, Frye R, Prince HM (2011). Phase 2 trial of romidepsin in patients with peripheral T-cell lymphoma. Blood.

[6] Rai S, Kim WS, Ando K (2023). Oral HDAC inhibitor tucidinostat in patients with relapsed or refractory peripheral T-cell lymphoma: phase IIb results. Haematologica.

[7] Kim WS, Fukuhara N, Yoon DH (2023). Darinaparsin in patients with relapsed or refractory peripheral T-cell lymphoma: results of an Asian phase 2 study. Blood Adv.

[8] Reyes C, Engel-Nitz NM, DaCosta Byfield S (2019). Cost of disease progression in patients with chronic lymphocytic leukemia, acute myeloid leukemia, and non-Hodgkin's lymphoma. Oncologist.

[9] Maddox JM, Horan M, Tafesh L (2021). DECC (dexamethasone, etoposide, chlorambucil, lomustine) as an oral chemotherapy regimen in relapsed and refractory diffuse large B-cell lymphoma. Br J Haematol.

[10] Vasanthamohan L, Parker B, Mangel J (2019). The use of CEP (lomustine, etoposide and prednisone), an all-oral palliative chemotherapy regimen, in aggressive lymphomas. Blood.

[11] Maybury B, Kimpton G, Otton S (2019). A retrospective multicentre study of COCKLE, an oral chemotherapy regimen, as palliative treatment for high grade lymphoma. Br J Haematol.

[12] Coleman M, Martin P, Ruan J (2008). Prednisone, etoposide, procarbazine, and cyclophosphamide (PEP-C) oral combination chemotherapy regimen for recurring/refractory lymphoma: low-dose metronomic, multidrug therapy. Cancer.

[13] Cullen M, Steven N, Billingham L (2005). Antibacterial prophylaxis after chemotherapy for solid tumors and lymphomas. N Engl J Med.

[14] Coleman M, Ruan G, Elstrom RL (2012). Metronomic therapy for refractory/relapsed lymphoma: the PEP-C low-dose oral combination chemotherapy regimen. Hematology.

[15] Bulley SJ, Santarsieri A, Lentell IC (2022). Managing relapsed refractory lymphoma with palliative oral chemotherapy: A multicentre retrospective study. EJHaem.

[16] Okamoto T, Nishimura Y, Yamada S (2000). Long-term administration of oral low-dose topoisomerase II inhibitors, MST-16 and VP-16, for refractory or relapsed non-Hodgkin's lymphoma. Acta Haematol.

[17] Dang NH, Pro B, Hagemeister FB (2007). Phase II trial of denileukin diftitox for relapsed/refractory T-cell non-Hodgkin lymphoma. Br J Haematol.

[18] Maruyama D, Tsukasaki K, Uchida T (2019). Multicenter phase 1/2 study of forodesine in patients with relapsed peripheral T cell lymphoma. Ann Hematol.

[19] Maruyama D, Nagai H, Maeda Y (2017). Phase I/II study of pralatrexate in Japanese patients with relapsed or refractory peripheral T-cell lymphoma. Cancer Sci.

[20] Barta SK, Gong JZ, Porcu P (2019). Brentuximab vedotin in the treatment of CD30+ PTCL. Blood.

